# Survival of bronchiectatic patients with respiratory failure in ICU

**DOI:** 10.1186/1471-2466-7-17

**Published:** 2007-12-10

**Authors:** Abdulaziz H Alzeer, Mohammed Masood, Syed Jani Basha, Shaffi A Shaik

**Affiliations:** 1 Division of Pulmonology, Department of Medicine, King Khalid University Hospital, Riyadh, Saudi Arabia; 2 Department of Family and Community Medicine King Khalid University Hospital, Riyadh, Kingdom of Saudi Arabia

## Abstract

**Background:**

The outcome of patients with bronchiectasis during and after their stay in the intensive care unit (ICU) has seldom been reported in the literature. Managing these patients in the ICU can be challenging because of the complex nature of their disease. This study aims to identify the in-hospital and long-term outcome of patients with bronchiectasis and respiratory failure (RF) in ICU.

**Methods:**

A retrospective study was carried out by studying all bronchiectatic patients admitted to the medical ICU for RF over a 10-year period (1995–2004).

**Results:**

The mean (± standard deviation) age of 35 patients was 63.5 ± 11.7 years and APACHE score was 22.3 ± 7.3. The 4-year mortality was 60%. Among the variables observed, age > 65 years (hazard ratio (HR): 4.15; 95% confidence interval (CI): 3.2–5.1), APACHE II score > 24 (2.6, 95% CI 1.7–3.5), intubation (2.81, 95 %CI 1.9–3.7), inotropic support (2.9, 95% CI 2.0–3.7), Home-O_2_ (4.0, 95% CI 2.7–5.2) and activity index (4.0, 95% CI 2.8–5.3) were associated with diminished survival in univariate analysis by Cox regression. By long rank test, survival probabilities were significantly low at these strata. Multivariate analysis of Cox proportional hazard model showed that age > 65 years (HR: 5.4, 95% CI 1.9–15.7); activity index (HR: 4.8, 95% CI 1.4–16.6); and inotropic support (HR: 3.8, 95% CI 1.5–10.1) were independently associated with reduced survival.

**Conclusion:**

The decreased survival of ICU patients was associated with age > 65 years, activity index (bedridden or wheelchair-bound) and use of inotropic support.

## Background

Bronchiectasis is an abnormal dilatation of the bronchial wall that is generally caused by infection [1]. Recurrent respiratory symptoms are common; however, the incidence of advanced bronchiectasis is now declining. Most patients are treated with a combination of postural drainage, antibiotics, and inhalation therapy [2,3]. Some patients need resectional surgery, which may cure the disease [4]. However, some patients may require intensive care therapy for acute respiratory failure (RF). Such patients may present with advanced RF and require assisted ventilation. Managing these patients in the intensive care unit (ICU) can be challenging because of the complex nature of their disease. Excessive sputum production, significant airway inflammation [5,6], and a change of chest wall geometry make their weaning from mechanical ventilation a burdensome process that is usually complicated with severe sepsis and multi-organ dysfunction syndrome (MODS). The ICU and long-term outcomes of these patients have not been fully analyzed. Moreover, the role of quality of life thereafter in the outcome for those who were successfully discharged has not been defined. We therefore decided to study the outcome of patients with bronchiectasis and chronic RF who require ICU admission, as well as factors that influence mortality, particularly the functional status of these patients.

## Methods

### Patients

This study was conducted in the medical ICU of King Khalid University Hospital, Riyadh, Saudi Arabia. The charts of all patients who were admitted with the diagnosis of bronchiectasis between January 1996 and January 2004 were reviewed. The study was approved by the Deanship of Scientific Research of King Saud University and the Research Ethics Committee of King Khalid University Hospital, Riyadh, Saudi Arabia. All patients were diagnosed by high-resolution CT scan (HRCT) based on previously reported criteria and had no limitations or withdrawal from therapy [7]. Patients were excluded if they had cystic fibrosis, were younger than 14, or were admitted for reasons other than RF.

Demographic data were age, gender, history of smoking, childhood infections, history of mycobacterium tuberculosis (TB) exposure, and long-term therapy of home oxygen (Home-O2). Symptoms of acute exacerbation such as shortness of breath, cough, sputum production, haemoptysis, and wheezes were included. Interventions that included mechanical ventilation (MV), inotropic support, and duration of ICU stay were recorded. Patients who underwent non-invasive mechanical ventilation (NIMV) were generally conscious with disturbed respiratory physiology. Puritan Bennett 7200 or Bennett 840 was used by applying two pressure levels [pressure support and positive end expiratory pressure (PEEP)]. Acidemic patients (pH < 7.25 and PCO_2 _> 55 mmHg) and those with unstable haemodynamics were intubated and ventilated with a volume targeted mode. Previous spirometry was measured with standard protocol recommended by the European Respiratory Society [8]. Obstructive ventilatory impairment is characterized by a low forced expiratory volume/s (FEV_1_) with relatively normal forced vital capacity (FVC), reduction of FEV_1_, FVC. FEV_1_/FVC % indicates mixed obstructive-restrictive ventilatory impairment.

### Assessment of severity

The worst physiological variable in the first 24 hours was used to calculate the Acute Physiology and Chronic Health Evaluation (APACHE II) score [9]. Premorbid dyspnea was scored according to the American Thoracic Society (ATS) scale [10]. The activities of daily living were recorded as 0 = working; 1 = independent (fully ambulatory and living without any assistance); 2 = restricted (able to live on their own and leave their homes to perform basic tasks, but severally limited in exercise ability); 3 = housebound (cannot leave their homes unassisted or leave their homes rarely, able to perform self-care but unable to do heavy chores such as house cleaning, cannot live alone, and may be institutionalized; and 4 = bedridden or wheelchair-bound.

### Outcome and long-term follow-up

The outcome is defined as survival and discharge from hospital. The charts of those who were successfully discharged were reviewed over a period of 4 years. When the follow-up was lost, the principal investigator contacted the patient's relatives. If the patient was deceased, the exact date of death was recorded.

### Statistical analysis

Descriptive statistics (mean and standard deviation) were calculated for all continuous variables. Survival was calculated from the data of patients who either survived up to 4 years or died within 4 years of being discharged from the hospital. Log-rank test was used to compare the survival pattern between the two strata of study variables. Univariate Cox regression was used to identify factors that were significantly associated with lower survival. The Cox proportional hazards model was used to identify the independent predictors associated with reduced survival. Patients who survived to the end of the follow-up period were considered censored. The analysis was done with SPSS pc+ version 10.0 software; *p *< 0.05 was considered to be statistically significant.

## Results

The 35 adults with bronchiectasis were studied retrospectively over 4 years. Mean age was 63.5 ± 11.7; 21 (60%) were females. All patients were in respiratory failure needing assisted ventilation. Cough was prevalent in 29 (82.8%) patients, sputum production was reported in 26 (74.3%), and 10 (29.9%) had haemoptysis. The etiology of bronchiectasis was found in 17 (48.6%) patients based on x-ray findings of old TB and 6 of them had received antituberculous chemotherapy. Twenty-five patients (71.4%) were treated with long-term Home-O_2 _therapy. Activity of daily living was identified as bedridden or wheelchair-bound in 24 (68.6%) cases and 8 (22.8%) patients were independent or restricted. CT scans demonstrated that four lobes or more were involved in 12 cases (34.3%) and that at least three lobes were involved for 5 (14.3%) patients.

Spirometry results were available for 23 (65.7%) patients. The mean FEV_1 _was 2.01 ± 0.46 L/s. The physiological status of the patients at ICU admission is reported in Table 1. Eleven patients (31.4%) were intubated before being admitted to the ICU or within the first day after their admission. In 4 patients (11.4%), PaO_2 _while breathing room air was < 60 mmHg. PaCO_2 _for 23 patients (65.7%) was > 55 mmHg or pH < 7.30 while on oxygen therapy. Diabetes mellitus was noted in 8 patients (22.8%); hypertension was present in 9 patients (25.7%), 7 patients (20%) had renal disease; liver disease was present in 9 patients (25.7%) and 6 (17%) were smokers.

**Table 1:** Variables of 35 patients with bronchiectasis at ICU admission.

**Variables**	**Mean ± S.D.**
Age (years)	63.5 ± 11.7
APACHE II score	22.3 ± 7.3
pH	7.3 ± 0.1
PaO_2 _(mmHg)	66.9 ± 34.1
PaCO_2 _(mmHg)	73.5 ± 25.1
FEV (L/s)	2.0 ± 4.6

### Risk factors for mortality in the ICU and after ICU stay

NIMV was used for 20 patients (57.1%). Eleven patients (31.4%) required intubation. Twelve (34.3%) died during ICU stay; all these had required intubation. ICU stays were 6.7 ± 7.3 days for survivors (range 1–28 days) and 14.5 ± 15.3 days for non-survivors (range 1–50 days). The mean FEV_1 _for survivors was 2.9 ± 0.6 and 0.9 ± 0.3 L/s for non-survivors. The median follow-up was 240 days (range 1–1,460 days). Cumulative mortality was 34.3% (*n *= 12) in ICU and 60% (*n *= 21) at 4 years. The causes of in-hospital deaths were: in 6 patients, complications of RF and MODS; in 2, myocardial infarction; in 1, presumed pulmonary embolism; and in 3, undetermined. The risk factors for hospital mortality by univariate analysis are shown in Table 2. Gender did not differentiate patient outcomes after ICU discharge. Survival decreased for patients who were older than 65, had an APACHE score > 24, were intubated, and who needed inotropic support, Home-O_2_, and were bedridden or chair-bound. Comparisons by the log-rank tests were significant (age > 65, *p *< 0.01; inotropic support, *p *= 0.01; and activity index, *p *= 0.01).

**Table 2:** Risk Factors for hospital mortality (by univariate analysis).

**Variables**	**HR (95% C.I)**	***p*-value**
Male gender	1.9 (0.7, 4.9)	0.12
Age > 65 years	4.1 (3.2, 5.1)	< 0.01
APACHE II Score > 24	2.6 (1.7, 3.5)	0.03
Intubation	2.8 (1.9, 3.7)	0.02
Inotropic support	2.8 (2.0, 3.7)	0.02
Home-O_2_	4.0 (2.7, 5.2)	0.02
Activity Index	4.0 (2.8, 5.3)	0.03

**Figure 1:**
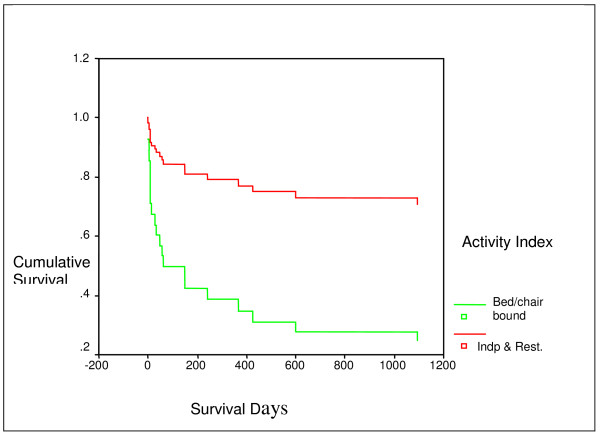
Survival curve for two categories of activity index using Cox model.

The results of the Cox proportional hazards analysis of factors associated with increased mortality after the ICU admission for RF are shown in Table 3. We included the dichotomous variables in multivariate analysis as patients attained a *p*-value of 0.5: age (> 65 vs. ≤ 65); APACHE score (> 24 vs. ≤ 24); intubation required (yes/no); inotropic support (yes/no); use of Home-O_2 _(yes/no); activities of living (bedridden or wheelchair-bound vs. working, independent, and restricted). Age > 65 (HR, 5.4; 95% CI, 1.9 to 15.7), inotropic support (HR, 3.8; 95% CI, 1.5, 10.1), and activity index (HR, 4.8; 95% CI, 1.4, 16.6) were independently associated with reduced survival. The Cox model survival curve for two categories of activity index is provided in Fig. 1, which indicates poor survival for patients who were bed/chair bound when compared with patients who were independent and restricted.

**Table 3:** Results of Cox Proportional Hazard Model analysis of the risk of death after the ICU admission for Respiratory Failure among 35 patients with Bronchiectasis.

**Predictor variables**	**HR**	**95% C.I.**	***p*-value**
Age > 65 years	5.4	(1.9, 15.7)	0.002
Inotropic support	3.8	(1.5, 10.1)	0.006
Activity Index	4.8	(1.4, 16.6)	0.013

## Discussion

The studies of long- and short-term outcome of bronchiectasis are scarce in the medical literature [11-13]; the clinical studies of patients managed in ICU are even fewer. A literature search found only one other study that evaluated ICU survival of patients with bronchiectasis [11]. This is likely because researchers concentrated on chronic obstructive pulmonary disease (COPD), which is more common than bronchiectasis in western countries. Bronchiectasis has recently declined, but still occurs in developing countries [14,15]. The in-hospital mortality in this group was 34.3%; and 4-year cumulative mortality was 60%. The major factors that influenced in-hospital mortality and subsequent death were age > 65, inotropic support, and premorbid level of activities.

In a recent retrospective study, DuPont *et al*. reported the mortality of their patients with bronchiectasis. Nineteen percent died in ICU and the 1-year mortality rate was 40%. Our findings on mortality are worse than they reported because the populations studied were mostly in advanced stages of the disease. Seventy-eight percent of our patients were using home O_2 _and 57% required NIMV compared to 25% and 27%, respectively, in the DuPont study [11]. Moreover, cylindrical bronchiectasis was seen in 56% of their patients whereas in our study, we found that 78% of our patients had cystic bronchiectasis, which suggests that our patients had more severe disease. Ashour suggested that cystic changes can worsen in pulmonary haemodynamics and more deterioration in gas exchange compared to cylindrical changes [16].

Univariate and multivariate analysis showed that premorbid level of activity was the independent determinant of outcome among our patients. This association has not been examined previously in bronchiectatic patients. Previous studies have shown functional status to be an important predictor of outcome for patients with COPD and RF, especially at 1 year or long term follow-up [17,18]. Based on these findings, patients with bronchiectasis who develop acute RF, require ICU admissions, and have poor functional status tend to have chronic progressive disease after their discharge. Their long-term prognosis is dismal.

Our results are also in accordance with the findings of Symonds *et al*., who conducted a prospective study of domiciliary nasal intermittent positive pressure ventilation in hypercapnic RF and found that only 20% of patients with bronchiectasis survived after 2 years compared to more favorable results in patients with scoliosis, previous poliomyelitis, or COPD [19].

In another study, Keistinen *et al*. reviewed the National Hospital Discharge Register in Finland and reported that patients with bronchiectasis had a more favorable outcome than those with COPD, but a less favorable outcome than asthmatics [12]. Variations between these studies may be ascribed to differences in severity of disease and disparity in population studied. The finding of low quality of life in this study highlighted important opportunities for improved care. For example, pulmonary rehabilitation, particularly postural drainage and behavioral therapy, appear to improve the quality of life in patients with COPD [20,21]. It has been shown also recently that, pulmonary rehabilitation is effective in improving exercise tolerance in bronchiectatic patients [22]. Selective patients may inhale antibiotics to reduce bacterial colonization, which will eventually reduce the probability of infection [23]. Moreover, immunization or vaccination in this high-risk group is important to prevent or minimize exacerbation.

Age was a statistically significant determination of 1-year and 4-year survival in this study. The association of age with long-term mortality risk could be modulated by other diseases and the severity of the acute illness. The abnormal physiology and the complications that develop during ICU stay strongly influence the patient's outcome. APACHE II score has been tried as a model of predictive survival in general ICU, but has not previously been evaluated in bronchiectatic patients. In this study, APACHE II score > 24 (*p *= 0.03) was associated with diminished survival in univariate analysis by lot regression. Moreover, the mean FEV_1 _for survivors was 2.9 ± 6 compared to 0.9 ± 0.3 for non-survivors.

Mechanical ventilation was associated with in-hospital mortality for our patients (Table 2). However, it was not an independent predictor of long-term mortality with multivariate analysis. The increased mortality of intubated patients was likely due to high APACHE II score. The in-hospital and long-term survival were also poor for patients who required inotropic support. This suggests that their haemodynamic instability was likely due to sepsis or other reasons such as cor pulmonale, which could eventually affect the left ventricular function [24]. Systematic cardiac function was not evaluated in this cohort.

The study has several inherent limitations. Because of the retrospective review of a prescreened population, the results may be biased by selection. Some results were missing or incomplete, which could have an impact on our statistics. Moreover, systematic nutritional assessment, which could affect mortality, was not performed. Finally, the population studied was relatively small to enable us to draw a short conclusion.

## Conclusion

We found that in-hospital and long-term outcome of patients with bronchiectasis can be predicted by taking in to account, age, interventions in the ICU, and severity score. We observed poor functional status, which may reflect the poor physical reserve in these patients.

## List of abbreviations

APACHE Acute Physiology and Chronic Health Evaluation

ATS American Thoracic Society

CI Confidence Interval

COPD Chronic Obstructive Pulmonary Disease

FEV1 Forced Expiratory Volume in 1 second

FVC Forced Vital Capacity

HRCT High Resolution Computerized Tomography Scan

ICU Intensive Care Unit

MODS Multi-organ Dysfunction Syndrome

NIMV Non-Invasive Mechanical Ventilation

RF Respiratory Failure

HR Hazard Ratio

TB Tuberculosis

## Competing interests

The author(s) declare that they have no competing interests.

## Authors' contributions

AHA – principal author and investigator. He initiated the study. He prepared the study proposal, recruited study patients and supervised the entire conduct of the study from beginning to preparation and submission of the manuscript.

MM – co-investigator. He helped in the recruitment of patients. He was instrumental in the collection of data, data entry, and literature review. Helped in the preparation and review of the manuscript.

SJB – co-investigator. He was instrumental in the review of the collected data. Together with the principal investigator and co-investigators, they ensured completeness of the data. Helped in the preparation and review of the manuscript.

SAS – reviewed the data and did the statistical analysis using appropriate statistical methods.

All authors read and approved the final manuscript.

## Pre-publication history

The pre-publication history for this paper can be accessed here:


